# Technological Alloying Impact on Formation of Phase Composition of Al-Fe-Si-X Alloys

**DOI:** 10.3390/ma18092096

**Published:** 2025-05-02

**Authors:** Violetta Andreyachshenko, Lenka Kunčická

**Affiliations:** 1 Abylkas Saginov Karaganda Technical University, Karaganda 100027, Kazakhstan; 2 Department of Metallurgical Technologies, Faculty of Materials Science and Technology, VŠB—Technical University of Ostrava, 17. listopadu 2172/15, 708 00 Ostrava, Czech Republic; 3 Faculty of Mechanical Engineering, Institute of Manufacturing Technology, Brno University of Technology, Technická 2896, 616 00 Brno, Czech Republic

**Keywords:** AlFeSi, intermetallic phases, simulation and modeling, diagrams phase transformation, microstructure

## Abstract

Given by their low weight and favorable combination of properties, Al-Fe-Si-based intermetallic and duplex alloys are widely used in mechanical engineering. The use of aluminum scrap for their production imparts the necessity for a thorough study of the impacts of presence of impurity/alloying elements on the phase composition. By this reason, individual impacts of the impurity/alloying elements present in the majority of commercial alloys on phase compositions of the alloys were studied herein. Particular emphasis was on the formation of the α phase and features of the α↔β transformation, as well as on their effects on the solidus, liquidus, and phase transformation temperatures. Modeling was used to study the synergistic effect of the simultaneous introduction of 12 elements into aluminum. According to the results, magnesium, copper, and nickel have a tendency to form combined intermetallic phases, and beryllium, as a structurally free element, forms precipitates even at minimum concentrations. Verification of the modelled results was performed using a real alloy prepared experimentally from commercially available raw materials. The comparison of the results provided by computer modeling and the actual phase composition showed sufficient agreement. The herein acquired results contribute to a deeper understanding of the features of phase transitions occurring during alloying of aluminum alloys and will also be useful for predicting microstructures and phase compositions of intermetallic alloys. This research has potential to inspire further development in materials science and engineering.

## 1. Introduction

Al-Si alloys are widely used in mechanical engineering. The produced components are typically made from alloys fabricated by casting (e.g., conventional casting in metal coquilles [1,2], vacuum arc melting [3], etc.), advantageously followed by a heat treatment [4,5]. However, to increase their durability and improve the mechanical properties, powder metallurgy [6,7]; additive manufacturing (e.g., selective laser melting [8,9,10], direct energy deposition [11,12], etc.); or deformation processing via conventional methods, e.g., rolling [13,14], as well as unconventional techniques, such as the versatile method of rotary swaging [15,16,17,18]; multidirectional forging [19,20]; or severe plastic deformation (SPD) [21,22], e.g., equal channel angular pressing (ECAP) [23,24,25] and related methods (e.g., ECAP with partial back pressure [26,27]), high pressure torsion (HPT) [28,29], or their combinations [30], can be applied.

For the Al-Si alloy system, iron is a non-avoidable impurity, especially if aluminum scrap is used as the raw material [31,32]. Therefore, Al-Fe-Si alloys are subject to ever-lasting comprehensive research to rationalize controlling their properties exhibited under specific operating conditions [33,34]. For example, Lan et al. [35] optimized the temperature stability of Al-Fe-Si alloy by varying the cooling rates, as well as iron and silicon contents. The improvement of mechanical performance of Al-Fe-Si alloys via application of various types of ECAP processing was studied, too [36,37,38]. The effect of various impurities is typically considered with the minimum amount of both iron and silicon [39,40]. Nevertheless, with an excess of the primary elements of the system, the impact of the impurities will differ. The need to consider Al-Si alloys enriched with iron is mentioned in a number of works, e.g., [41,42]. However, the iron content remains low.

The majority of the contemporary studies investigated this particular alloy system by setting up experiments with the use of pure components [43,44]. Due to the ever-increasing demand for processing of aluminum alloys and, consequently, their increasing contamination with impurities other than silicon and iron, it is important to consider that the contemporary fabricated aluminum alloys non-negligibly contain impurities. Therefore, their effects on the phase composition of Al-Fe-Si alloys need to be examined. It is known that alloying with various elements has a significant effect on the phase composition of Al-Fe-Si alloys and, therefore, on its microstructure, properties, and overall performance [45,46,47]. Transition metals can advantageously be introduced into the composition to transform the α hexagonal phase into a cubic modification, as well as to suppress the α↔β transition to impart greater plasticity [48]. Simultaneously, approaching the casting temperature to the liquidus temperature can also change the morphology of the primary phase in the sense of its coarsening [49].

The interest of scientists in Al-Fe-Si alloys is ever-lasting, and a large number of research works have been published in recent years. Nevertheless, understanding of the occurring phase transformations is still limited due to the absence of fundamental studies of the individual and synergistic effects of impurity/alloying elements on the final phase compositions. The phase formation features of the system under consideration can lead to erroneous identification of phases based solely on their atomic composition. The main identification errors are caused by the closeness of the atomic composition of the intermetallic binary phases with dissolved impurity elements formed by partial substitution of iron atoms, for example, in the theta phase and ternary intermetallic phases. The latter, in turn, are also capable of dissolving a certain amount of impurity elements. Among the primary prospects of this study is to help researchers conducting practical studies of the system using identification techniques using SEM/TEM microscopy with microanalyzers and will contribute to minimizing erroneous phase identification. The applicability of the results to a wide range of alloys using flexible modeling conditions in the ThermoCalc software package is expected to be wide, too.

Within the present study, the effects of the presence of impurity/alloying elements, most probably occurring within the majority of the common grades of aluminum alloys, on the phase transformations were studied (primarily using computer modeling). The effects of the elements were evaluated upon adding each element separately. To assess the mutual influence of the possibly occurring impurity elements, a case of simultaneous introduction of all the impurities was also considered. To verify the computed results, experimental alloys corresponding to real commercial alloys were finally prepared and examined. The uniqueness of the work primarily lies in the use of a high-iron and high-silicon fully intermetallic alloy with the composition that is as close as possible to the composition of the α phase. The composition of the α phase was chosen according to the hypothesis of obtaining an intermetallic alloy that is capable of undergoing plastic deformation for the prospective purpose of forming.

## 2. Materials and Methods

### 2.1. Basics for Modelling

Selecting the basic composition of the Al-Fe-Si system was done on the basis of a model construction of the phase composition for an alloy with the constant aluminum content (60%) and a variable composition of silicon and iron, with the condition Σ (Fe + Si) = 40% being met. After determining the most attractive area of study of the alloy, the possibility of expanding the area of the α phase was assessed, i.e., the most favorable ferrous intermetallic phase that binds iron and silicon by alloying and microalloying. The main levels of impurity contents (all in wt.%) in aluminum grades, adopted in accordance with SS 4784-2019 aluminum and wrought aluminum alloys, are given in Table 1. Based on the data in Table 1, it is clear that the maximum amount of silicon of 13% contained the silumins, the maximum amount of iron of 2% was within the Al-Fe system alloys, contents of 7% of copper and 1.4% of nickel were in the Al-Cu-Mg and Al-Cu-Mn systems, and a content of manganese of 1.5% was in the Al-Mn system. The amount of magnesium reached to 7% for the Al-Mg system, while the amount of zinc was regulated up to 9% for the Al-Zn-Mg system. Chromium, titanium, vanadium, zirconium, and beryllium were present in almost all the systems, while their maximum volumes were 0.35%, 0.2%, 0.15%, 0.45%, and 0.005%, respectively. Thus, the specified content was for each case taken as the maximum possible content of alloying/impurity elements, when processed alloys were used as starting materials in synthesizing the Al-Fe-Si alloys. In this research, impurities contained in steels were not considered.

Using the ThermoCalc software package with the TCAL8: Al-Allous v8.2 database (software version 2024a, Solna, Sweden), a phase diagram for the three-component base alloy was modeled; the ranges of occurrence of individual phases, phase transformation temperatures, and liquidus and solidus temperatures were determined. Then, four-component alloys were modeled to assess the individual impact of each impurity element. Further, a multicomponent system was modeled to assess the mutual impact of the elements under consideration.

First of all, the constant element and variable elements were determined to assess the individual impact of each element on the phase composition of the alloy. Considering the fact that impurities form compounds with aluminum or dissolve therein, additional elements were introduced when constructing the diagrams by reducing the amount of aluminum in the alloy, while silicon and iron were additionally introduced to achieve the planned compositions. For the modeling, the transition of elements from the original components to the alloy was assumed to be 100%. The main principles taken into account during modeling in ThermoCalc software can be summarized via the following relations (Equations (1)–(3)):

(1) dU=Q+W

(2) dU=dQ−PdV

(3) G=U−TS+PV

where U is internal energy, P is pressure of the environment, V is volume, T is absolute temperature of the environment, and S is entropy. T and V controlled outside (external variables).

At equilibrium (Equations (4) and (5)):

(4) U=f(T,V)

(5) $$dU={\left(\frac{\partial U}{\partial T}\right)}_{V}dT+{\left(\frac{\partial U}{\partial V}\right)}_{T}dV$$

and combined with Equation (2) (Equations (6)–(8)):

(6) $$dQ={\left(\frac{\partial U}{\partial T}\right)}_{V}dT+{\left(\frac{\partial U}{\partial V}\right)}_{T}dV+PdV$$

(7) $$dQ={\left(\frac{\partial U}{\partial T}\right)}_{V}dT+\left({\left(\frac{\partial U}{\partial V}\right)}_{T}+P\right)dV$$

(8) $$C_{V}={\left(\frac{\partial U}{\partial T}\right)}_{V}$$

Heterogeneous equilibrium in a system consisting of N phases and K components occurs under the following conditions (Equation (9)):

(9) $$\left.\begin{array}{c} \begin{array}{c} T1=T2\ldots \ldots \ldots \ldots =TN-1=TN\\ P1=P2\ldots \ldots \ldots \ldots =PN-1=PN\\ \ldots \ldots \ldots \ldots \ldots \ldots \ldots \ldots \ldots \ldots \ldots \ldots \ldots \ldots \ldots \ldots .. \end{array}\\ \mu_{1}^{1}=\mu_{2}^{1}=\ldots \;\ldots \;\ldots \;\ldots =\mu_{N-1}^{1}=\mu_{N}^{1}\\ \mu_{1}^{K}=\mu_{2}^{K}=\ldots \;\ldots \;\ldots \;\ldots =\mu_{N-1}^{K}=\mu_{N}^{K} \end{array}\right\}$$

The chemical potential of a component in a phase depends on its concentration (Equation (10)):

(10) $$\mu_{i}=\mu_{0}+kT\ln(\gamma C_{i})$$

where μ_0_ is the chemical potential of the i-component in the standard state, k is the Boltzmann constant, γ is the activity coefficient of the i-component in the phase, and C_i_ is the concentration of the i-component in the phase.

The variability of a system consisting of K components and N phases, when only pressure P and temperature T act as external parameters, is equal to (Equation (11)):

(11) $$C=\left(K-1\right)N+2-K\left(N-1\right)$$

Gibbs phase rule (Equation (12)):

(12) C=K−N+2

In cases where the number of external parameters is reduced by one (P = const or T = const), the phase rule is used in the following form (Equation (13)):

(13) C=K−N+1

If the external parameters are constant (i.e. P = const and T = const), the phase rule has the following form (Equation (14)):

(14) C=K−N

To describe phase equilibria in the ThermoCalc environment, a graphical method of description was further used, i.e., phase equilibria diagrams were plotted. The following boundary conditions were used in the modeling: Temperature 1200 °C, Pressure 100,000 Pa, System size 1 mol, Composition (see Table 2).

The direction of the reactions of interaction of the elements under consideration was determined by the magnitude of the oxidation potential, which in turn depends on the temperature, concentration of the elements, and their place in the series of activity of the elements. When the number of phases and the type of phases in the system changed, the ratio of the elements changed, which in turn led to a change in the direction of the reactions in the system.

The phase volume fractions in the simulation were determined by the lever rule implemented using the ThermoCalc software for the characteristic points of the phase diagrams under consideration. When comparing with the experimental data, the phase volume fractions were calculated by analyzing the microstructure using the image analysis tools available in SEM.

### 2.2. Experimental Verification

For the experimental study of the results of the computational modeling of the phase composition, 12 model alloys with constant concentrations of iron (33%) and silicon (7%) and variable concentrations of aluminum and impurity/alloying elements, as well as 2 experimental alloys synthesized using commercial raw materials, were selected.

To verify the acquired results, an alloy was synthesized by the method of electric arc surfacing under a flux grade layer AN-348 (fused and sintered). For these purposes, special packages were prepared, which were alternating layers of aluminum and silicon. For the process, we used commercial aluminum alloy AD31N as the initial material. The AD31N alloy has a small number of impurities of magnesium, iron, silicon, and zinc. It is a commercial duralumin typically used in the form of 3 mm thick rolled sheets. We used specifically the commercial alloy to ensure compliance of the practically used alloy with the parameters presented for the theoretical alloys used in the modeling assessing the influences of the individual elements. Silicon was introduced into the system in the form of powder placed between the aluminum layers. The initial powder of fraction −500 μm was additionally ground in a vibratory grinder to fraction −63 μm. The accuracy and uniformity of grinding was controlled by rubbing through a sieve with a cell size of 63 μm. After grinding, a colloidal aqueous solution was prepared from the silicon powder at a ratio of 2 parts of powder to 1 part of water. The solution was then applied to aluminum sheets with weight control for precise dosing of the amount of silicon. The sheets with applied silicon were successively combined into packages of 4 sheets per package and dried at room temperature for 24 h. Further, the source of iron in the alloy was a steel consumable electrode made of rolled sheet steel grade St3 with a thickness of 2 mm. The iron-containing component and crystalline silicon were used in the following proportions: aluminum alloy 60 g; steel electrode 30 g; pre-crushed silicon 5, 7, and 10 g. The electrodes were cut to the dimensions of 30 × 150 mm, and the working length of the electrode participating in the synthesis was 100 mm. The electric arc initiated by the source ensured melting of all the three components. The uniformity of the composition was ensured by the alternate arrangement of aluminum and silicon, and simultaneous mixing during melting ensured formation of phases in accordance with the phase composition characteristic of a given composition at the corresponding temperature. At the same time, laminar motion of micro-volumes of the molten components and directional natural heat dissipation led to vertical directionality of the final microstructure of the ingot. The exclusion of evaporation of components and protection from interaction with the environment was ensured by the use of flux AN348. The features of the process are described more in detail in [50].

After fabricating the ingot, the chemical composition was determined using an X-ray fluorescence analyzer Vanta Element-S (Olympus Europa SE & Co. KG, Hamburg, Germany) with the possibility to detect light elements. Then, based on the data acquired from the real alloy, a refined phase diagram was constructed. In the “as prepared” state, the microstructure was analyzed using an optical microscope (Olympus Europa SE & Co. KG, Hamburg, Germany), and the phases were identified using the EDS analysis on a scanning electron microscope (SEM by Tescan Group, a.s., Brno, Czech Republic). The microstructure analysis of the sample was carried out, taking into account the homogeneity analysis of various microstructures with a comparison of the upper, central, lower, and peripheral layers of the billet. The microstructure showed satisfactory homogeneity with a slight gradient along the height of the ingot. The presented photographs of the microstructure are shown for the central layers of the billet. Microhardness was measured using a Wilson VH1150 device (Buehler, Lake Bluff, IL, USA) to determine the mechanical properties under compression. Finally, cylindrical samples with a diameter of 10 mm and length of 20 mm were prepared and tested using an Instron 5982 equipment (Illinois Tool Works Inc., Glenview, IL, USA).

## 3. Results and Discussion

### 3.1. Modelling

**Figure 1:**
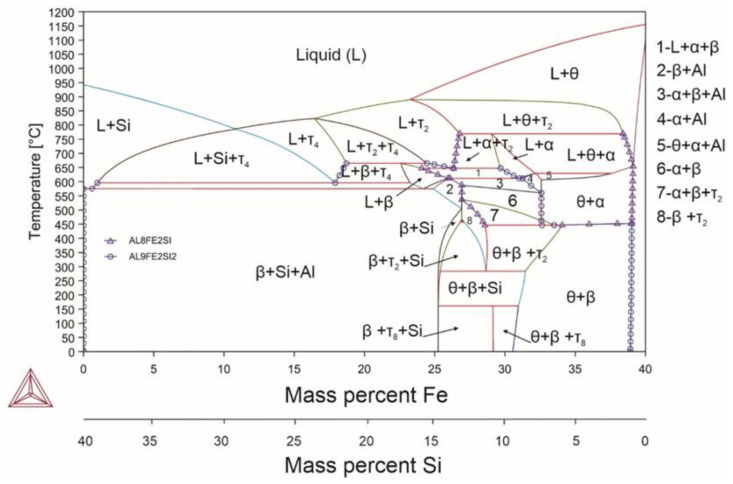
Phase diagram for alloy 0 (Al_60_Fe_33_Si_7_).

Figure 1 shows the basic phase diagram for the three-component Al-Fe-Si system that demonstrated mutual impact of silicon and iron on the phase composition of the alloy with the aluminum content of up to 60%, with the total amount of silicon and iron of 40%.

It is generally known that phases featuring crystal lattices with low-symmetry are difficult to process by plastic deformation, and alloys featuring such phases exhibit particularly low plasticity, e.g. [51,52]. On the other hand, materials with a highly symmetric crystal lattices, such as the cubic and hexagonal ones, are capable of undergoing relatively large plastic deformations and demonstrate fairly good plasticity, e.g. [53,54,55,56,57,58,59,60]. Based on these facts, the areas of the diagram characterized by FCC solid solution of aluminum and/or the α phase with the hexagonal crystal lattice are of interest. With the aluminum content of 60%, different iron and silicon ratios revealed nine zones with the α phase and five zones with FCC aluminum. There were no homogeneity areas for the α phase [61]. In this case, one of the regions consisted entirely of FCC aluminum and the α phase. It is worth noting that the widest range of compositions including iron from 0 to 25% and the corresponding amount of silicon (40-Fe)% ensured the presence of FCC aluminum from 570 °C up to the room temperature. The α phase is a high-temperature phase; its minimum temperature is 430 °C, and then it transforms to β phase characterized by a large content of iron and a lower content of aluminum and silicon. In general, the α phase does not form at silicon contents exceeding 17%; however, absence of silicon and excess of iron also complicate its formation. The upper temperature limit of the existence of the α phase is 770 °C. The solidus temperatures for a high-silicon alloy and a high-silicon and high-iron alloy are 600 °C and 620 °C, respectively.

The β and θ phases are considered to be harmful phases for aluminum alloys due to their morphology. These phases typically have acicular structures leading to degradation of mechanical properties. In some cases, they are represented by block inclusions. However, for alloys that consist mainly of intermetallic compounds, the morphology of such phases changes completely and can be represented by large blocks or a duplex microstructure. In this case, silicon can easily be incorporated into the θ phase, controlling multi-stage phase transitions to the other intermetallic phases [62]. Based on the above-mentioned information, it is particularly interesting to study the alloy in the region consisting of two phases, α and FCC aluminum, while assessing the impact of alloying/impurity elements on the resulting phase composition. At the same time, given the extremely narrow range of the two-phase region limited to 2%, it is more appropriate to use the alloy of composition region 5 (Figure 1) with its subsequent transition to a duplex alloy to identify the impact of impurity/alloying elements. The alloys under consideration are very sensitive to chemical composition. Therefore, for deeper understanding, Figure 1 shows the change in phase composition with varying amounts of iron and silicon within this range. For an alloy with the Al_60_Fe_33_Si_7_ composition, the solidus temperature was 629 °C, and the α↔β transformation occurred at the temperature of 446 °C.

First of all, for deeper understanding of the effect of alloying, the effect of alloying with each impurity element was considered separately. Considering that in the studied case, the alloying/impurity elements entered the alloy together with aluminum, they were introduced into the calculation at the expense of aluminum with the base amounts of iron and silicon of 33% and 7%, respectively. The amount of the impurity element was found, taking into account the total aluminum content of 60%, with the assumption of complete transition of the charge materials into the alloy composition. The use of such an assumption is legitimate due to the use of flux, as well as the approximate transition coefficient for the selected method of 90–95% for all three base components.

Let us consider in detail the phase transformations for the selected alloy composition. When cooling, the alloy underwent the following reactions (Equations (15)–(21)):

(15) $$L\overset{1070℃}{\to }L+\theta$$

(16) $$L+\theta \overset{880℃}{\to }L+\theta +\tau_{2}$$

(17) $$L+\theta +\tau_{2}\overset{769℃}{\to }L+\theta +\alpha$$

(18) $$L+\theta +\alpha \overset{629℃}{\to }\theta +\alpha +Al$$

(19) $$\theta +\alpha +Al\overset{610℃}{\to }\theta +\alpha$$

(20) $$\theta +\alpha \overset{446℃}{\to }\theta +\beta +\tau_{2}$$

(21) $$\theta +\beta +\tau_{2}\overset{376℃}{\to }\theta +\beta$$

That is, the primary phase crystallizing from the liquid was the θ phase, which continued to separate from the liquid as the temperature decreased with an increase in the volume fraction to a temperature of 880 °C. Then, during the interaction of the θ phase and the liquid, the τ_2_ phase was formed. The amount of the θ phase decreased due to the formation of the τ_2_ phase up to 769 °C. At this temperature, phase recrystallization of the τ_2_ phase to the α phase occurred. Then, the interaction of the θ phase and the liquid continued; the amount of both phases decreased; and, simultaneously, the α phase was formed. The maximum amount of the α phase, which remained constant in the temperature range of 607–570 °C, was 93.68%. This was accompanied by an almost complete exhaustion of the θ phase. Then, an insignificant decrease in the amount of the α phase by 1% was observed up to the temperature of 446 °C.

As the liquid became depleted in silicon and iron, the remainder of the liquid crystallized as almost pure aluminum at the temperature of 629 °C. However, as the temperature further decreased, a reaction between aluminum and the θ phase occurred, forming the α phase. This was accompanied by an increase in the volume fraction of the latter and complete depletion of the FCC of aluminum. At the temperature of 446 °C, the α phase decomposed to form the β and θ phases. Despite the fact that it is customary to consider the transformation as α↔β, in fact, it was the decomposition of the α phase that was observed, forming an almost equal amount of the β (50%) and θ (45%) phases. The remaining 5% was the τ_2_ phase, which then dissolved at the temperature of 376 °C, increasing the amount of both the phases. With further cooling, the quantitative ratio of the β/θ phases did not change. Table 2 shows the compositions of the alloys studied in this work.

Since the elements are herein introduced into the alloy directly from aluminum, it is advisable to evaluate the impact of these elements from the point of view of reducing the amount of aluminum at the expense of introducing an alloying/impurity element.

Copper is one of the most common elements present in all the groups of aluminum alloys, and its content is typically very high compared to the other elements. It is noteworthy that high copper impurities are characteristic for all the groups, not only for copper-based systems (Al-Cu-Mg, Al-Cu-Mn).

**Figure 2:**
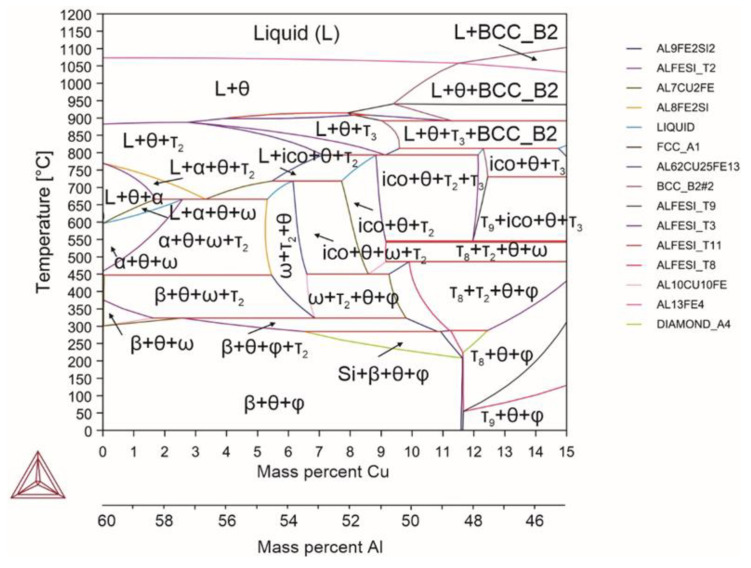
Phase diagram for alloy 1 (Al_55.8_Fe_33_Si_7_Cu_4.2_).

The four-component phase diagram of Al-Fe-Si-Cu (Alloy 1) shows that even an insignificant presence of copper in the alloy promoted the formation of the tetragonal ω phase, which is an Al_7_Cu_2_Fe intermetallic compound. Copper was also present in dissolved form in the liquid, and in an insignificant amount in the θ phase. The type of crystal lattice of the ω phase predicted its brittleness but at the same time high hardness. Laplanche et al. [63] noted a transition from brittle to plastic behavior at temperatures between 700 and 750 °C. Particles of the ω phase in aluminum alloys have a plate-like shape and a tendency to precipitate along grain boundaries [64], which likely decrease the mechanical properties (Figure 2).

The reactions that occurred when 4.2% copper was introduced were as follows (Equations (22)–(29)):

(22) $$L\overset{1070℃}{\to }L+\theta$$

(23) $$L+\theta \overset{900℃}{\to }L+\theta +\tau_{11}$$

(24) $$\tau_{11}\overset{900{}^{\circ}C}{\to }\tau_{3}\overset{898{}^{\circ}C}{\to }\tau_{2}$$

(25) $$L+\theta +\tau_{2}\overset{690℃}{\to }L+\theta +\tau_{2}+\omega$$

(26) $$L+\theta +\omega +\tau_{2}\overset{665℃}{\to }\alpha +\theta +\omega +\tau_{2}$$

(27) $$\alpha +\theta +\omega +\tau_{2}\overset{447℃}{\to }\beta +\theta +\omega +\tau_{2}$$

(28) $$\alpha +\theta +\omega +\tau_{2}\overset{323℃}{\to }\beta +\theta +\varphi +\tau_{2}$$

(29) $$\beta +\theta +\varphi +\tau_{2}\overset{310℃}{\to }\beta +\theta +\varphi$$

Introducing small amounts of copper contributed to decreasing the solidus temperature down to 600 °C, but increasing the copper content contributed to increasing the solidus temperature, and in the range from 2% to 5% of copper, the solidus temperature was ~660 °C. Further increase in the amount of copper contributed to an even greater increase in the solidus temperature with an expansion of the τ_2_ phase region. However, given that increasing the amount of copper was achieved by reducing the total amount of aluminum, this effect was probably complex (mutual with the impact of the total amount of iron in the alloy). The presence of copper also increased the temperature of the α↔β transformation, with the α phase ceasing to exist already at 5% of copper, while the β phase was detected at up to 11.5% of copper. The ω↔φ transformation temperature was 323 °C. The φ hexagonal phase can, on the contrary, have an opposite effect on the mechanical properties of the alloy.

The limited area of the α phase was associated with the limited solubility of copper in the solid solution. The θ phase was capable of dissolving of up to 0.5 mole fraction of copper (1 mass fraction), which led to its precipitation as an intermetallic ω phase upon cooling. Further increase in the amount of copper at the expense of aluminum led to the formation of Al_65_Cu_20_Fe_15_ quasicrystalline, which binds mainly aluminum, as well as iron and copper. The simultaneous coexistence of the ico-phase and α-phase is impossible; at the same time, the ico-phase, similar to the ω-phase, is a high-temperature phase and transforms into a number of other intermetallic phases with decreasing temperature. Introducing copper promoted an increase in the α↔β transformation temperature to 447 °C. In general, the addition of copper led to increasing plasticity due to the presence of the φ hexagonal phase at temperatures below 323 °C.

**Figure 3:**
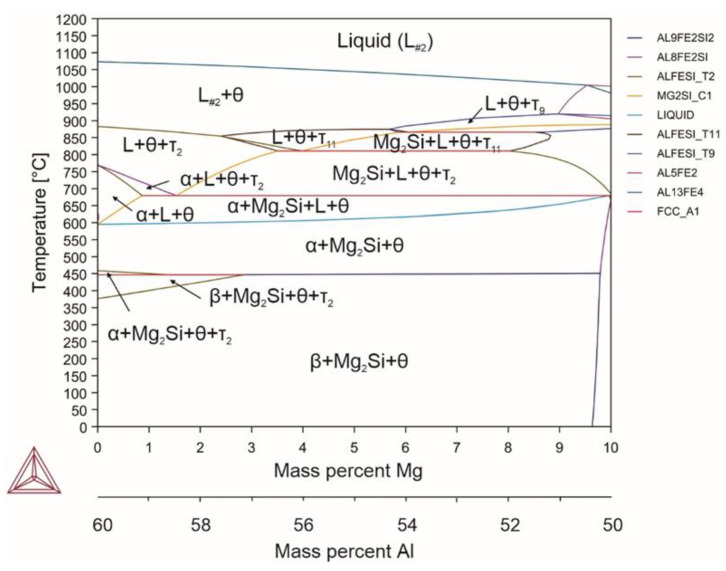
Phase diagram for alloy 2 (Al_59.16_Fe_33_Si_7_Mg_0.84_).

When magnesium (alloy 2) was introduced, magnesium silicide was formed, i.e., the cubic Mg_2_Si compound, a strengthening phase in aluminum alloys (Figure 3). Magnesium additives did not affect the α↔β transformation temperature. However, they significantly reduced both the solidus temperature (by more than 30 °C) and upper limit of the α phase precipitation (by 50 °C). At temperatures above the solidus, magnesium completely dissolved in small quantities and precipitated into an independent phase with the content of more than 1.5%.

Reactions occurring in the system (Equations (30)–(36)):

(30) $$L\overset{1060℃}{\to }L+\theta$$

(31) $$L+\theta \overset{870℃}{\to }L+\theta +\tau_{2}$$

(32) $$L+\theta +\tau_{2}\overset{720℃}{\to }L+\theta +\tau_{2}+\alpha$$

(33) $$L+\theta +\alpha +\tau_{2}\overset{670℃}{\to }\alpha +L+\theta +{Mg}_{2}Si$$

(34) $$\alpha +L+\theta +{Mg}_{2}Si\overset{600℃}{\to }\alpha +\theta +{Mg}_{2}Si$$

(35) $$\alpha +\theta +{Mg}_{2}Si\overset{446℃}{\to }\beta +\theta +{Mg}_{2}Si+\tau_{2}$$

(36) $$\beta +\theta +{Mg}_{2}Si+\tau_{2}\overset{400℃}{\to }\beta +\theta +{Mg}_{2}Si$$

Magnesium can dissolve only in a liquid; intermetallic phases do not dissolve magnesium, similarly as magnesium silicide does not dissolve iron and aluminum atoms. In general, as magnesium forms a compound with silicon, the proportion of the θ phase in the alloy increases. The amount of the α phase decreases with increasing magnesium content. With the magnesium content of 6% at 500 °C, the proportion of the α phase was 35.68%, as opposed to 74.76% with 2% of magnesium. Magnesium also increased the β/θ ratio at room temperature, which led to the prevalence of the θ phase with its content of up to 75%. This effect of magnesium is characteristic for contents of magnesium of up to 10%. For example, Jin et al. [65] noted that increasing the magnesium content suppressed the transformation from α-AlFeSi to β-AlFeSi. However, increasing the silicon content promoted the transformation of α-AlFeSi to β-AlFeSi. It is obvious that the high amount of silicon, and especially the high amount of iron, led to the absence of this effect when introducing magnesium in the present study.

Reactions occurring in the system (Equations (37)–(46)):

(37) $$L\overset{1070℃}{\to }L+\theta$$

(38) $$L+\theta \overset{880℃}{\to }L+\theta +\tau_{2}$$

(39) $$L+\theta +\tau_{2}\overset{810℃}{\to }L+\theta +\tau_{2}+\alpha_{Mn}$$

(40) $$L+\theta +\tau_{2}+\alpha_{Mn}\overset{762℃}{\to }\alpha +L+\theta +\alpha_{Mn}$$

(41) $$\alpha +L+\theta +\alpha_{Mn}\overset{680℃}{\to }\alpha +\theta +\alpha_{Mn}$$

(42) $$\alpha +\theta +\alpha_{Mn}\overset{530℃}{\to }\alpha +\theta +\alpha_{Mn}+\tau_{2}$$

(43) $$\alpha +\theta +\alpha_{Mn}+\tau_{2}\overset{507℃}{\to }\alpha +\alpha_{Mn}+\theta_{\#2}+\tau_{2}$$

(44) $$\alpha +\theta +\theta_{\#2}+\alpha_{Mn}+\tau_{2}\overset{462℃}{\to }\alpha +\theta_{\#2}+\theta +\beta +\tau_{2}$$

(45) $$\alpha +\theta_{\#2}+\theta +\beta +\tau_{2}\overset{450℃}{\to }\theta_{\#2}+\theta +\beta +\tau_{2}$$

(46) $$\alpha +\theta_{\#2}+\theta +\beta +\tau_{2}\overset{310℃}{\to }\theta_{\#2}+\theta +\beta$$

**Figure 4:**
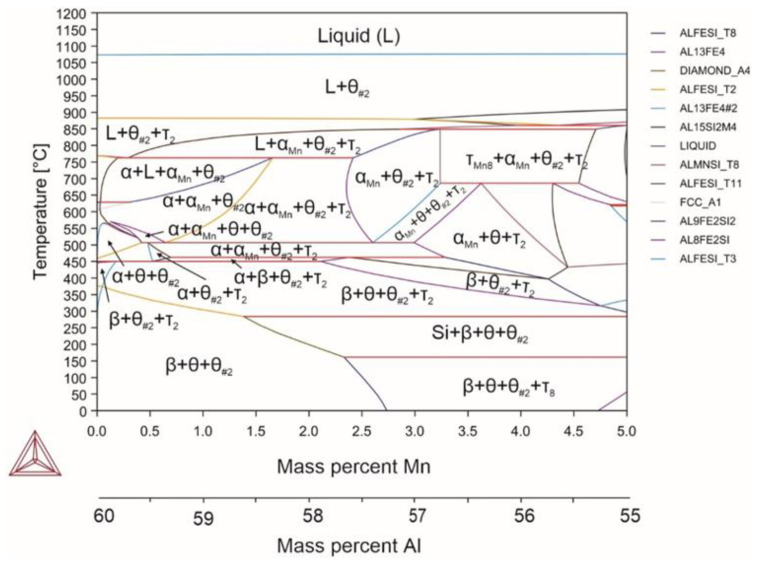
Phase diagram for alloy 3 (Al_59.1_Fe_33_Si_7_Mn_0.9_).

When introducing manganese to the Al-Fe-Si alloy (Alloy 3), it was capable of replacing the hexagonal α phase with the cubic lattice α phase (Figure 4). It is known that the effective role of manganese is observed with a manganese content of up to 0.5 [66]. However, when introducing manganese at the expense of aluminum, the hexagonal α phase ceased to form already at the manganese content of 2.6%, i.e. in terms of the ratio of Fe/Mn < 0.1. Manganese did not have any fundamental effect on the α↔β temperature and does not change the liquidus temperature at all. It caused solidus temperature increase by 51 °C. Moreover, by increasing the manganese content to more than 3.2%, an increase in the solidus temperature to 850 °C was observed.

The amount of the α phase was noticeably reduced compared to the base composition (by approximately 20–30% depending on the temperature) already with introducing 1% manganese. The maximum amount of the cubic phase was only 22.15%. With introducing manganese, two modifications of the θ phase were formed. One of them was virtually no different from the similar phase in the alloy with the base composition and contained up to 12% of silicon, and the second phase modification contained up to 5% of dissolved manganese. An increased amount of silicon in the θ phase can lead to incorrect identification of this phase. The manganese content of more than 2.6% led to the formation of the τ_8_orthorhombic intermetallic phase containing an increased amount of silicon (manganese was absent in this phase).

**Figure 5:**
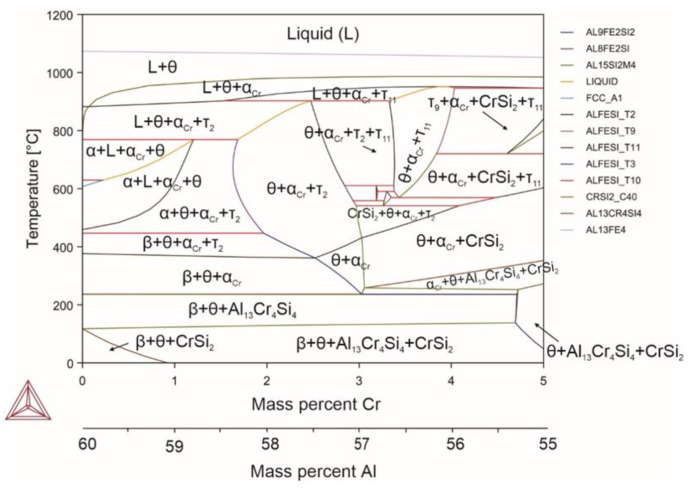
Phase diagram for alloy 4 (Al_59.79_Fe_33_Si_7_Cr_0.21_).

Chromium, similar to manganese, promotes the transformation of αh into αc [67], significantly reducing the temperature limit of the modified phase to 250 °C. The amount of αc is proportional to the amount of chromium introduced. Complete displacement of the αh phase with the αc phase was observed with the introduction of 1.8% of chromium at the expense of aluminum. When chromium was added, the αc homogeneity region was absent and chromium silicide formed at room temperature, i.e., below 100 °C (it is worth noting that its formation was also observed at high temperatures with increasing the chromium amount over 3%). Despite the fact that chromium somewhat reduced the liquidus temperature when introduced at the level of impurity content in aluminum alloys, chromium did not affect all the other temperature transitions. On the other hand, with increasing the chromium content over 1.2%, a significant increase in the solidus temperature up to 900 °C was observed (αh is not formed in this case, Figure 5).

Reactions occurring in the system (Equations (47)–(56)):

(47) $$L\overset{1070℃}{\to }L+\theta$$

(48) $$L+\theta \overset{910℃}{\to }L+\theta +\alpha_{Cr}$$

(49) $$L+\theta +\alpha_{Cr}\overset{880℃}{\to }L+\theta +\alpha_{Cr}+\tau_{2}$$

(50) $$L+\theta +\alpha_{Cr}+\tau_{2}\overset{769℃}{\to }L+\theta +\alpha +\alpha_{Cr}$$

(51) $$L+\theta +\alpha +\alpha_{Cr}\overset{629℃}{\to }\theta +\alpha +\alpha_{Cr}$$

(52) $$\theta +\alpha +\alpha_{Cr}\overset{460℃}{\to }\theta +\alpha +\alpha_{Cr}+\tau_{2}$$

(53) $$\theta +\alpha +\alpha_{Cr}+\tau_{2}\overset{446℃}{\to }\theta +\beta +\alpha_{Cr}+\tau_{2}$$

(54) $$\theta +\beta +\alpha_{Cr}+\tau_{2}\overset{380℃}{\to }\theta +\beta +\alpha_{Cr}$$

(55) $$\theta +\beta +\alpha_{Cr}\overset{236℃}{\to }\theta +\beta +{Al}_{13}{Cr}_{4}{Si}_{4}$$

(56) $$\theta +\beta +{Al}_{13}{Cr}_{4}{Si}_{4}\overset{90℃}{\to }\theta +\beta +Cr{Si}_{2}$$

The effect of chromium at the temperatures of 600 °C and 300 °C is of particular interest. Despite the visible tendency of chromium to increase the αc phase content, there was no homogeneity zone for this phase, and its maximum volume fraction was 35%. With increasing the amount of chromium, the αc phase was replaced by the Al_13_Cr_4_Si_4_ phase. A distinctive feature of chromium is its non-solubility in all the intermetallic phases of the ternary system. Chromium is found exclusively in chromium-containing intermetallics and chromium silicide.

**Figure 6:**
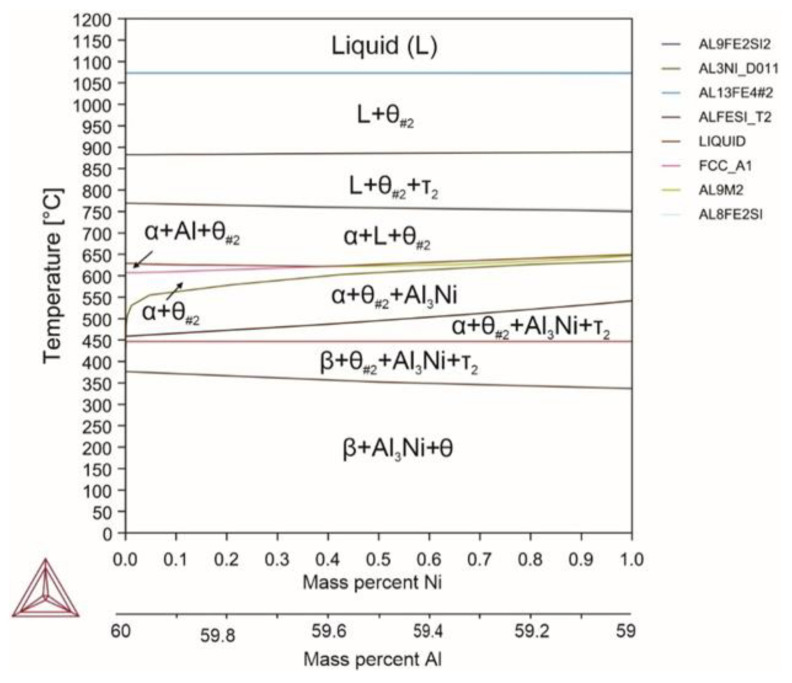
Phase diagram for alloy 5 (Al_59.16_Fe_33_Si_7_Ni_0.84_).

Nickel can dissolve in the θ phase, but its main part is bound into nickel aluminide. An exceptional feature of nickel is its ability to completely suppress the formation of the θ phase for an increased temperature range, from the liquid phase to 330 °C (Figure 6). With decreasing the temperature, the amount of nickel in the θ phase decreased sharply, which led to formation of the θ phase with an insignificant amount of dissolved nickel. Similar phenomena were observed by Elsharkawi et al. [68]. In addition, the β phase and nickel aluminide were present at room temperature. Nickel did not dissolve in the α and β phases and did not significantly affect the β/θ quantitative ratio. Its effect on the phase transition temperatures was also barely noticeable. It did not reduce the liquidus temperature, but it increased the solidus temperature by 12 °C, while the upper limit of the α phase formation decreased by 16 °C, which was accompanied by decreasing the volume fraction of the α phase, both near the upper limit (by 7%) and near the α↔β phase transition (by 18%).

Reactions occurring in the system (Equations (57)–(65)):

(57) $$L\overset{1070℃}{\to }L+\theta_{\#2}$$

(58) $$L+\theta_{\#2}\overset{880℃}{\to }L+\theta_{\#2}+\tau_{2}$$

(59) $$L+\theta_{\#2}+\tau_{2}\overset{753℃}{\to }L+\theta_{\#2}+\alpha$$

(60) $$L+\theta_{\#2}+\alpha \overset{641℃}{\to }\alpha +\theta_{\#2}+{Al}_{9}{Ni}_{2}$$

(61) $$\theta_{\#2}+\alpha +{Al}_{9}{Ni}_{2}\overset{640℃}{\to }\alpha +\theta_{\#2}$$

(62) $$\alpha +\theta_{\#2}\overset{620℃}{\to }\alpha +\theta_{\#2}+{Al}_{3}Ni$$

(63) $$\alpha +\theta_{\#2}+{Al}_{3}Ni\overset{520℃}{\to }\alpha +\theta_{\#2}+{Al}_{3}Ni+\tau_{2}$$

(64) $$\alpha +\theta_{\#2}+{Al}_{3}Ni+\tau_{2}\overset{446℃}{\to }\beta +\theta_{\#2}+{Al}_{3}Ni+\tau_{2}$$

(65) $$\beta +\theta_{\#2}+{Al}_{3}Ni+\tau_{2}\;\overset{350℃}{\to }\beta +\theta$$

**Figure 7:**
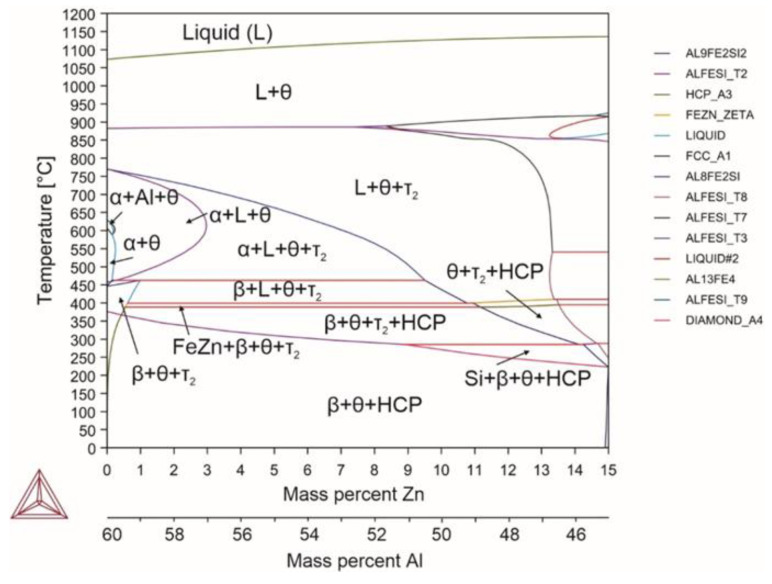
Phase diagram for alloy 6 (Al_54.6_Fe_33_Si_7_Zn_5.4_).

Unlike the other elements, zinc solubility in the phase components of the alloy was very high. Thus, having a low melting point, zinc significantly reduced the solidus temperature (Figure 7). With the zinc content of 5.4% due to the total aluminum content, the solidus temperature was only 399 °C, which was significantly lower than the solidus temperature of the base alloy, although the liquidus temperature was higher than the melting point of the base alloy (1110 °C). The temperature range for the α phase narrowed, the upper limit was 650 °C and the lower one was 462 °C, and the content of the α phase dropped almost twice. At the room temperature, the phase composition also depended significantly on the amount of zinc introduced. It began to precipitate in the form of individual zinc inclusions containing traces of the remaining alloy components, simultaneously changing the β/θ ratio from ~50/50 to an alloy consisting of 90% of the θ phase with 15% of zinc.

Reactions occurring in the system (Equations (66)–(72)):

(66) $$L\overset{1100℃}{\to }L+\theta$$

(67) $$L+\theta \overset{880℃}{\to }L+\theta +\tau_{2}$$

(68) $$L+\theta +\tau_{2}\overset{650℃}{\to }L+\theta +\alpha +\tau_{2}$$

(69) $$L+\theta +\alpha +\tau_{2}\overset{462℃}{\to }L+\theta +\beta +\tau_{2}$$

(70) $$L+\theta +\beta +\tau_{2}\overset{399℃}{\to }\theta +\beta +\tau_{2}+FeZn$$

(71) $$\theta +\beta +\tau_{2}+FeZn\overset{388℃}{\to }\theta +\beta +\tau_{2}+HCP$$

(72) $$\theta +\beta +\tau_{2}+HCP\overset{310℃}{\to }\theta +\beta +HCP$$

**Figure 8:**
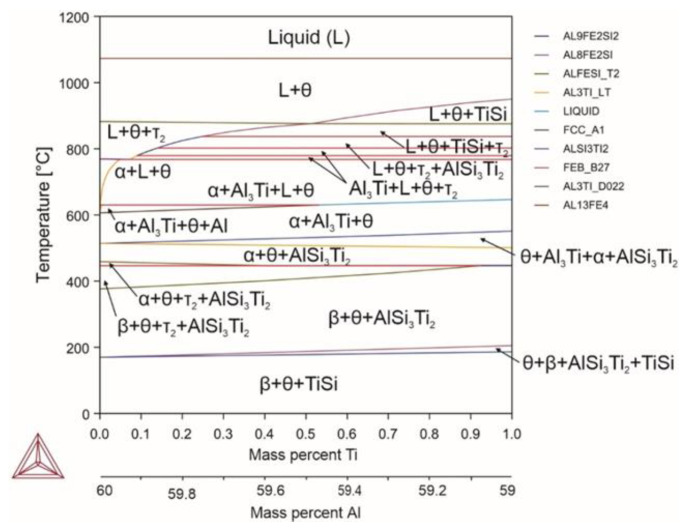
Phase diagram for alloy 7 (Al_59.88_Fe_33_Si_7_Ti_0.12_).

Titanium, despite its high melting point, did not change the liquidus and solidus temperatures of the system, nor the temperature limits of the α phase (Figure 8). Neither changed the amount of the α phase, compared to the base composition. At room temperature, titanium silicide was formed with the element ratio of 1:1 (the amount was proportional to the introduced titanium).

Reactions occurring in the system (Equations (73)–(82)):

(73) $$L\overset{1070℃}{\to }L+\theta$$

(74) $$L+\theta \overset{880℃}{\to }L+\theta +\tau_{2}$$

(75) $$L+\theta +\tau_{2}\overset{790℃}{\to }L+\theta +\tau_{2}+{Al}_{3}Ti$$

(76) $$L+\theta +\tau_{2}+{Al}_{3}Ti\overset{767℃}{\to }L+\theta +\alpha +{Al}_{3}Ti$$

(77) $$L+\theta +\alpha +{Al}_{3}Ti\overset{630℃}{\to }\theta +\alpha +{Al}_{3}Ti+Al$$

(78) $$\theta +\alpha +{Al}_{3}Ti+Al\overset{620℃}{\to }\theta +\alpha +{Al}_{3}Ti$$

(79) $$\theta +\alpha +{Al}_{3}Ti+Al\overset{513℃}{\to }\theta +\alpha +{AlSi}_{3}{Ti}_{3}$$

(80) $$\theta +\alpha +{AlSi}_{3}{Ti}_{3}\overset{446℃}{\to }\theta +\beta +\tau_{2}+{AlSi}_{3}{Ti}_{3}$$

(81) $$\theta +\beta +\tau_{2}+{AlSi}_{3}{Ti}_{3}\overset{390℃}{\to }\theta +\beta +{AlSi}_{3}{Ti}_{3}$$

(82) $$\theta +\beta +{\mathrm{AlSi}}_{3}{\mathrm{Ti}}_{3}\;\overset{171℃}{\to }\theta +\beta +TiSi$$

Titanium did not dissolve in any of the phases, and it formed the Al_3_Ti and AlSi_3_Ti_2_ compounds at elevated temperatures. The α phase can occur at titanium contents of up to 6.7%. In general, when titanium-containing compounds were formed, the amounts of available aluminum and silicon decreased, which led to an increased θ phase amount. Simultaneously, at temperatures below the temperature of AlSi_3_Ti_2_ phase into TiSi phase transformation, silicon was bound into titanium silicide, thereby releasing aluminum and iron atoms, which also contributed to increasing the proportion of the θ phase. Despite this, more favorable conditions were formed for the formation of β phase particles, resulting in the alloy consisting of 80–90% of β phase up to the introduction of 1% of titanium.

**Figure 9:**
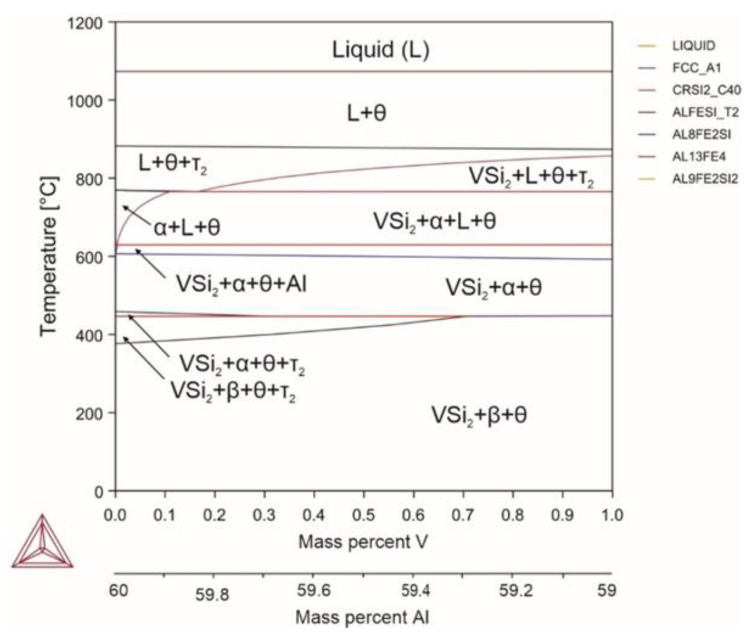
Phase diagram for alloy 8 (Al_59.91_Fe_33_Si_7_V_0.09_).

Similar to titanium, introducing vanadium in quantities of up to 1% did not significantly affect the temperatures of phase transitions of the system, with the difference being that vanadium, when introduced in the amount of less than 1%, did not form stable compounds with aluminum but bound silicon into the VSi2 compound that did not undergo transformations during cooling (Figure 9). At the same time, due to silicon binding, a narrow temperature region (629–610 °C) of the FCC aluminum solid solution was formed. With increasing amount of the introduced vanadium, the region of the FCC aluminum solid solution expanded and was able to reach to 503 °C, although the volume fraction of the aluminum solid solution was only 2.7%. Vanadium in a dissolved form was present only in the FCC aluminum solid solution, along with silicon and iron, and was not soluble in the intermetallic phases. A distinctive feature of vanadium is that it does not introduce additional phase transformations over the entire temperature range and does not change the temperatures of phase transitions.

Reactions occurring in the system (Equations (83)–(91)):

(83) $$L\overset{1070℃}{\to }L+\theta$$

(84) $$L+\theta \overset{880℃}{\to }L+\theta +\tau_{2}$$

(85) $$L+\theta +\tau_{2}\overset{767℃}{\to }L+\theta +\alpha$$

(86) $$L+\theta +\alpha \overset{750℃}{\to }L+\theta +\alpha +{VSi}_{2}$$

(87) $$L+\theta +\alpha +{VSi}_{2}\overset{629℃}{\to }Al+\theta +\alpha +{VSi}_{2}$$

(88) $$Al+\theta +\alpha +{VSi}_{2}\overset{610℃}{\to }\theta +\alpha +{VSi}_{2}$$

(89) $$\theta +\alpha +{VSi}_{2}\overset{450℃}{\to }\theta +\alpha +{VSi}_{2}+\tau_{2}$$

(90) $$\theta +\alpha +{VSi}_{2}+\tau_{2}\overset{446℃}{\to }\theta +\beta +{VSi}_{2}+\tau_{2}$$

(91) $$\theta +\beta +{VSi}_{2}+\tau_{2}\overset{390℃}{\to }\theta +\beta +{VSi}_{2}$$

**Figure 10:**
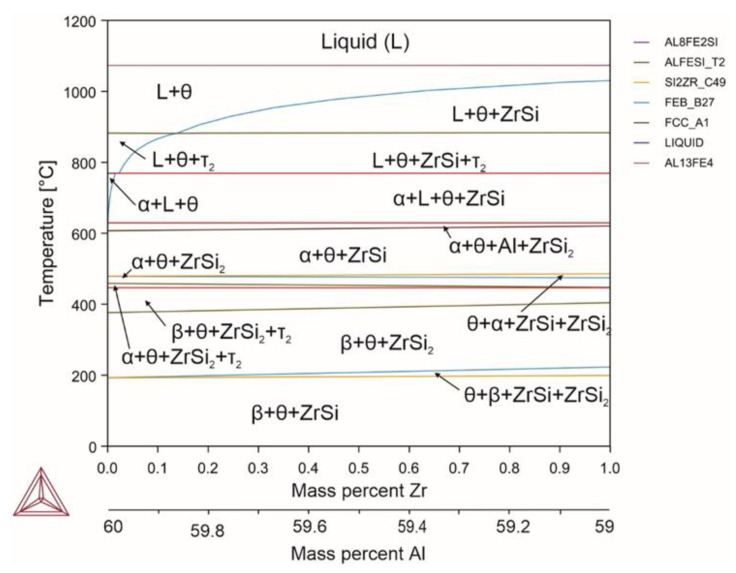
Phase diagram for alloy 9 (Al_59.73_Fe_33_Si_7_Zr_0.27_).

Zirconium is typically added to aluminum alloys to provide thermal stability by increasing the recrystallization temperature via formation of the Al3Zr compound with the zirconium content of 0.2–0.4% [69]. However, in the present study, the Al3Zr compound was not detected (Figure 10). This was probably caused by the increased amount of silicon and iron. When introducing up to 1% of zirconium, two types of silicides were formed: zirconium silicide, and zirconium bisilicide. Due to the formation and phase transformation of silicides, additional areas on the diagram were formed but they did not affect the basic phase components. Zirconium had zero solubility in intermetallic phases, and aluminum was completely absent in its silicides. However, traces of iron were able to be observed in both the silicide and in zirconium bisilicide. Zirconium did not affect the phase transition temperatures or the volume fraction of the α phase. The α phase was found in up to 10% zirconium.

Reactions occurring in the system (Equations (92)–(102)):

(92) $$L\overset{1070℃}{\to }L+\theta$$

(93) $$L+\theta \overset{930℃}{\to }L+\theta +ZrSi$$

(94) $$L+\theta +ZrSi\overset{880℃}{\to }L+\theta +ZrSi+\tau_{2}$$

(95) $$L+\theta +ZrSi+\tau_{2}\overset{769℃}{\to }L+\theta +\alpha +ZrSi$$

(96) $$L+\theta +\alpha +ZrSi\overset{629℃}{\to }Al+\theta +\alpha +ZrSi$$

(97) $$Al+\theta +\alpha +ZrSi\overset{620℃}{\to }\theta +\alpha +ZrSi$$

(98) $$\theta +\alpha +ZrSi\overset{480℃}{\to }\theta +\alpha +ZrSi+{Si}_{2}Zr$$

(99) $$\theta +\alpha +ZrSi+{Si}_{2}Zr\overset{450℃}{\to }\theta +\alpha +ZrSi+{Si}_{2}Zr+\tau_{2}$$

(100) $$\theta +\alpha +ZrSi+{Si}_{2}Zr+\tau_{2}\overset{446℃}{\to }\theta +\beta +ZrSi+{Si}_{2}Zr+\tau_{2}$$

(101) $$\theta +\beta +ZrSi+{Si}_{2}Zr+\tau_{2}\overset{390℃}{\to }\theta +\beta +ZrSi+{Si}_{2}Zr$$

(102) $$\theta +\beta +ZrSi+{Si}_{2}Zr\;\overset{200℃}{\to }\theta +\beta +$$

**Figure 11:**
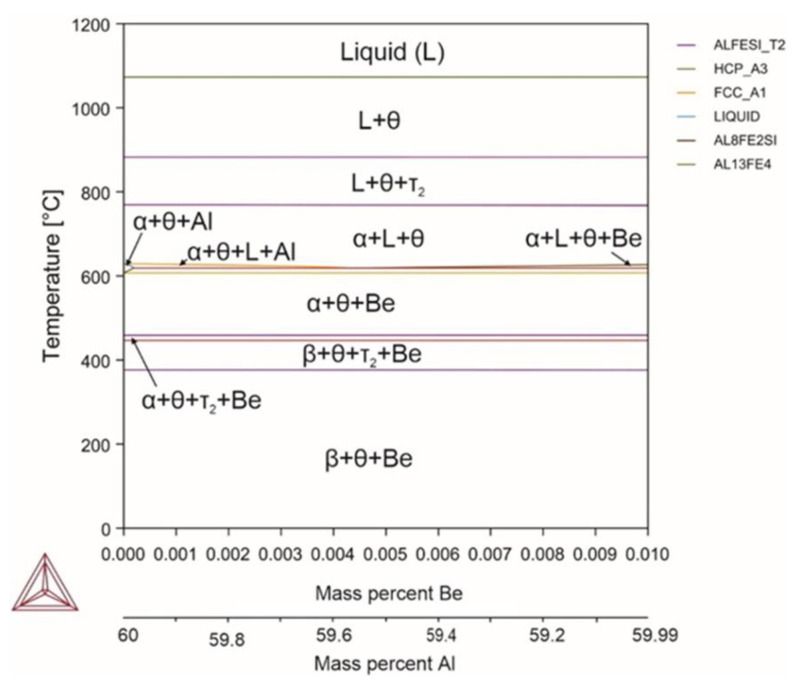
Phase diagram for alloy 10 (Al_59.997_Fe_33_Si_7_Be_0.003_).

Introducing beryllium reduced the liquidus temperature of the alloy by 7 °C and the solidus temperature by 9 °C (Figure 11). The temperature of the α↔β phase transformation did not change, nor did the volume fraction of the α phase. Beryllium, without dissolving into intermetallic phases, is able to dissolve aluminum, iron, and silicon. Beryllium forms structurally free precipitates, without changing the number of phase components.

The obtained results on the effects of the alloying/impurity elements on the phase composition, and accordingly the properties, show that not all the elements included in the composition of common industrial alloys dissolved in the basic phase components. Some elements, such as copper, manganese, chromium, and titanium, formed independent intermetallic inclusions. The addition of magnesium led to the formation of silicides; chromium, titanium, vanadium, and zirconium also formed silicides, while nickel formed aluminides. At elevated temperatures, zinc formed a compound with iron, but at room temperature, it precipitated as a structurally free element. Beryllium precipitated at any temperature, dissolving the other elements in small amounts.

Reactions occurring in the system (Equations (103)–(111)):

(103) $$L\overset{1070℃}{\to }L+\theta$$

(104) $$L+\theta \overset{880℃}{\to }L+\theta +\tau_{2}$$

(105) $$L+\theta +\tau_{2}\overset{768℃}{\to }L+\theta +\alpha$$

(106) $$L+\theta +\alpha \overset{620℃}{\to }Al+\theta +\alpha$$

(107) $$Al+\theta +\alpha \overset{618℃}{\to }Al+\theta +\alpha +Be$$

(108) $$Al+\theta +\alpha +Be\overset{610℃}{\to }\theta +\alpha +Be$$

(109) $$\theta +\alpha +Be\overset{450℃}{\to }\theta +\alpha +Be+\tau_{2}$$

(110) $$\theta +\alpha +Be+\tau_{2}\overset{446℃}{\to }\theta +\beta +Be+\tau_{2}$$

(111) $$\theta +\beta +Be+\tau_{2}\;\overset{380℃}{\to }\theta +\beta +Be$$

**Figure 12:**
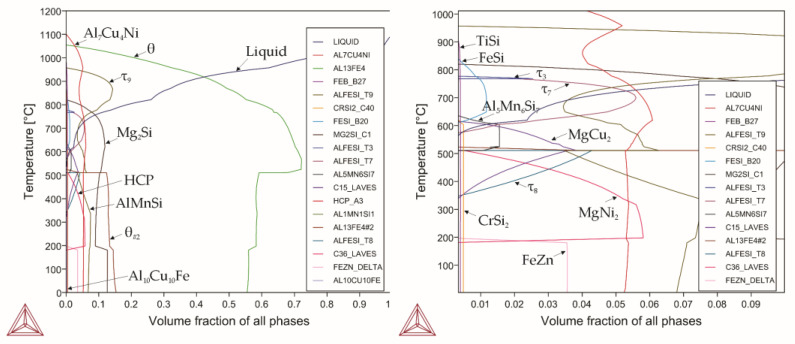
Phase diagram for alloy 11.

Obviously, the simultaneous presence of all the considered elements when using aluminum scrap is unlikely, especially with regard to the weight fraction of the impurity/alloying elements. However, when considering such a hypothetical possibility and conducting phase composition modeling simultaneously with all the considered elements, the resulting phase composition (Figure 12) shows that each element had a predominantly similar effect as when evaluated separately. It is worth noting that with the simultaneous introducing of all the elements, the amount of aluminum in the system reduced significantly to 43.77%. This introduces conditions at which the presence of the α phase is thermodynamically impossible. In addition, its cubic modifications formed due to the effects of chromium and manganese were absent, and the low-temperature β phase was also not formed. Manganese and chromium, present in industrial alloys of the AlSi system, are part of the intermetallic compound Al_15_Si_2_M_4_, where M is a combination of Fe, Cr, and Mn atoms; thus, the formed phase is written as Al_15_Si_2_(Fe,Cr,Mn)_4_. The intermetallic compound belongs to the cubic system. In this case, the formation of such harmful phases as θ and β is suppressed, which causes a favorable effect of alloying the AlSi system alloys with manganese and chromium both separately and with complex alloying with both elements. At the same time, when introducing these elements separately, they also form a cubic phase Al_15_Si_2_(Fe,Cr/Mn)_4_, designated as αc. But with the simultaneous introduction of all alloying elements, the formation of this cubic modification was not observed, and manganese and chromium were completely bound into intermetallic compounds (manganese into ternary intermetallic compounds, chromium forms chromium carbide).

Nevertheless, this calculation was performed to identify possible mutual interactions of the impurity elements. In the fractional composition diagram, two groups of the Laves phases formed during the interaction of magnesium with copper and nickel, i.e., cubic MgCu_2_ and hexagonal MgNi_2_. Decreasing the available amount of aluminum led to the formation of new intermetallic compounds: Al_7_Cu_4_Ni, as well as phases based on AlFeSi such as τ_3_, τ_7_, and τ_8_.

Moreover, increased activity and tendency of copper, magnesium, and nickel to form binary and ternary compounds were also observed. A similar tendency for impurity elements to interact was reported by Kotiadis et al. [70]; they also noted that the nature of this interaction and the formed phases were primarily influenced by the amount of available aluminum. At the same time, the volume fraction of these compounds was lower than 10%. The Al_7_Cu_4_Ni phase became primary, followed by the θ phase, which was formed upon supercooling by 40 °C, and its content exceeded 70% in the temperature range of 500–600 °C. The θ phase also became a competing phase at the temperature of 510 °C but with dissolved impurity elements. Thus, the alloy consisted of 70% of the θ phases with different stoichiometric compositions at the room temperature. The solidus temperature was 533 °C. The crystallization process ended at the moment of beryllium formation. It is noteworthy that most silicide-forming elements lost their ability to form them under the combined effect of the other elements, and they were found in dissolved forms in the other phases. Chromium silicide, magnesium silicide, and zirconium silicide were able to be found as separate inclusions. Beryllium also fell out as a structurally free element, copper-containing intermetallics, and an iron–zinc compound.

**Figure 13:**
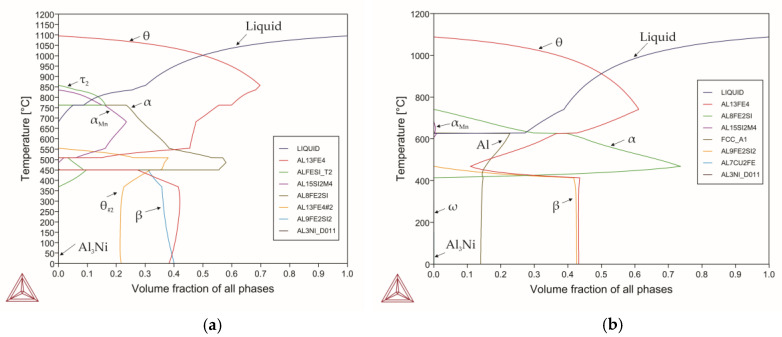
Phase diagram for (experimental) (**a**) alloy 12; (**b**) alloy 13.

The obtained data on the interaction of the individual elements included in the experimental theoretical alloy contribute to better understanding of the impact of certain components on the phase composition of real alloys and compounds. The phase interaction features are characteristic of aluminum alloys and can be successfully scaled up to industrial processes. This applies to both individual and synergistic effects of the elements. The possibility of scaling up is guaranteed by the used approach, i.e., analysis of the effect of impurity/alloying elements in the maximum amount that can be introduced when using secondary aluminum sources. To verify the presented model, a phase composition diagram of the experimental alloy was constructed using the flux synthesis (Figure 13). Comparison of the phase composition and microstructure of the alloys has the prospect to facilitate the process of phase identification.

As can be seen from the phase diagram, the features of the interaction of impurity/alloying atoms with each other and with the base elements of the system were identical to those of alloy 11, which has already been considered. Nickel did not react with manganese, which could have led to the formation of new phases. Manganese was completely incorporated into the ternary intermetallic compound, forming a cubic modification of the α phase, without forming any homogeneity zone. This led to the simultaneous coexistence of both the αh phase and αc phase. The formation of the αc phase led to a noticeable decrease in the volume fraction of the αh phase. As expected, this type of interaction was observed for both the experimental alloys.

For alloy 12, the temperature range of the αc phase precipitation was limited to 510 °C, which contributed to an increase in the amount of the αh phase, present up to the temperature of 450 °C, in the range of 510–450 °C. For alloy 13, the αc phase was formed in a smaller volume, since the manganese level in the alloy was significantly lower. This contributed to an increase in the volume of the αh phase and a decrease in the lower boundary of the phase to the temperature of 410 °C. The reduced silicon content led to crystallization of the remaining liquid in the form of FCC aluminum solid solution, in which iron and silicon were also dissolved. At room temperature, ~14% of FCC aluminum was retained.

It is worth noting that the number of β and θ phases in the alloy at room temperature was at a comparable level, ~40% of each. The presence of copper led to the formation of a ternary intermetallic compound—the ω phase. Nickel precipitated in the form of the Al_3_Ni compound in an amount proportional to the nickel content in the alloy.

**Figure 14:**
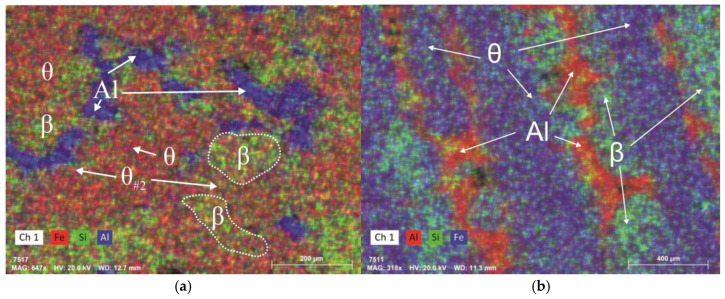
Element distribution map obtained by SEM for alloy 12 (experimental) (**a**) and alloy 13 (experimental) (**b**).

The total effect of impurities/alloying elements on the thermodynamic characteristics of the alloys is summarized in Table 3. The distribution map of elements on the surface of the studied section of the experimental alloy 12 is shown in Figure 14.

### 3.2. Experimental Analysis

The experimental alloys were characterized, as expected, by uniform microstructures along the width and height of the ingot. Figure 14a,b show that the microstructures of the experimental alloys were completely intermetallic, with the θ phase identified with the following composition: Al(61.08–62.91) Si(36.3–37.89); the θ#2 phase, in which up to 9% of silicon was dissolved; and the β phase. However, a solid solution of FCC aluminum, a product of phase transitions occurring during the α↔β transformation, was also present in small quantities; silicon and iron were not detected in FCC aluminum. However, the two prepared alloys featured a fundamental difference, caused by their chemical compositions, and consequently their phase compositions. In other words, despite the fact that the phase composition at room temperature was similar for both the alloys, the order of phase transitions during cooling determined the features of the morphology at room temperature. In the experimental alloy 12, the presence of manganese led to the formation of a cubic α phase at temperatures between 840 °C and 520 °C, with subsequent phase recrystallization. Such a phase transition changed the vertical direction of the structural components, as can be seen in Figure 14a. Figure 14b then shows the element distribution map for alloy 13, with FCC, θ, and β phases. In addition to the above-mentioned phase components, inclusions of nickel aluminide were found in the metal structure, which was in good agreement with the results of computer modeling. As expected, no additional intermetallic phases were detected.

**Figure 15:**
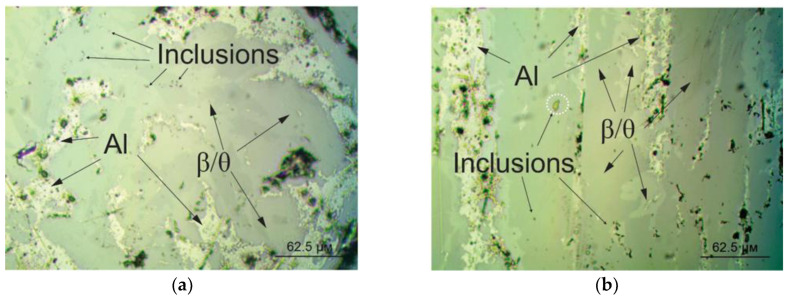
Microstructure of experimental alloys: (**a**) alloy 12; (**b**) alloy 13.

Figure 15a,b show the microstructures of the experimental alloys 12 and 13, acquired using an optical microscope. A comparative analysis of these two alloys confirmed that both the alloys were intermetallic. In both cases, the crystal growth direction coincided with the direction of heat dissipation. Despite the large proportion of FCC aluminum in alloy 13, the coagulating effect of manganese was clearly visible. It is worth noting that manganese additives did not completely suppress the α↔β transformation, but they contributed to a more favorable phase morphology, which was especially noticeable at the interphase boundaries. The amount of silicon present in alloy 13, which was insufficient for complete iron binding, contributed to an increase in the FCC proportion. The microstructure images show inclusions of Al_3_Ni, and for alloy 13, also phase ω (indicated by a circle).

The actual microstructure of the alloys, determined by their phase compositions, correlated well with the phase compositions predicted by modeling, which once again emphasizes the value of the results obtained in this work and the possibility of scaling up to real industrial processes. The nature of the morphology of the phase components determines the level of mechanical properties acquired during mechanical testing. The volume fraction of the FCC aluminum phase affected the microhardness especially. The use of the considered experimental alloys most clearly showed how high-temperature phase transformation can influence the final morphology of phase components, which, consequently, determines the level of material properties. The presence of a high proportion of intermetallic phases provides the mentioned phases with high microhardness values. Interlayers of regions consisting of FCC aluminum solid solution provide a damping effect, which manifests in favorable (for metal-ceramic materials) strength properties, especially in the direction normal to the direction of maximum heat dissipation during synthesis.

**Figure 16:**
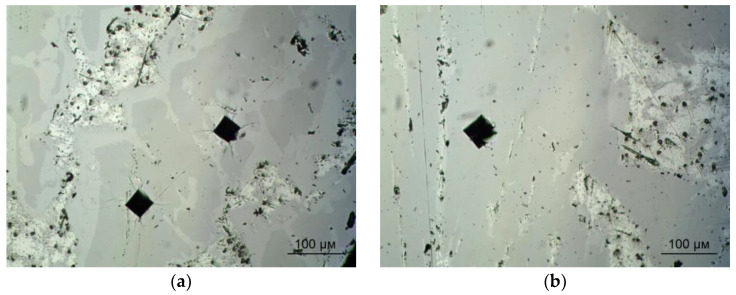
Microhardness measurement for experimental alloys: (**a**) alloy 12; (**b**) alloy 13.

The microhardness of the intermetallic phases of alloy 13 was 450 ± 11 HV1, while the microhardness of the aluminum solid solution was 133 ± 6 HV1. The microhardness of the intermetallic phases of alloy 12 then reached 756 ± 12 HV1, and the microhardness of the aluminum solid solution had a value close to that of alloy 13, i.e., 128 ± 6 HV1. Examples of indents performed for both the alloys are depicted in Figure 16a,b.

Last but not least, the compression test enabled us to determine the compressive strength and the degree of deformation that the alloys can withstand without failure. These values were 52 ± 2 MPa at the degree of deformation of 10%, and 49 ± 2 MPa at the degree of deformation of 11% for alloys 12 and 13, respectively. The experimentally acquired values of mechanical properties for both the prepared alloys are summarized in Table 4. The mechanical properties were determined in the “as-prepared” state. In addition, the mechanical properties are also supposed to be sensitive to the method of synthesis of the alloys and can have higher values for different methods, e.g. [71].

## 4. Conclusions

By applying computer modeling to assess the phase compositions, we clearly demonstrated phase formations in the intermetallic alloys of the Al-Fe-Si system with the addition of 12 most common elements characteristic for industrial aluminum alloys. The impact on the temperatures of phase transformations and the conditions of formation of the α intermetallic phase were characterized. It was found that none of the alloying elements increased the volume content of the α phase in the intermetallic alloy of the composition that is close to the composition of the α phase. The main impact of impurity/alloying elements is in the change in the phase transitions temperatures. For a simultaneous introduction of all the 12 investigated impurity/alloying elements, a synergistic effect on increasing the volume content of the α phase was also not found. Copper, nickel, and magnesium have the ability to interact and to form new phases, and when introduced together, they form two types of the Laves phase. However, in all the presented cases of alloying, the amount of the θ phase at room temperature increased, in some cases with phase modifications capable of dissolution of some alloying elements therein. Verification carried out using an experimental alloy showed sufficient agreement between the results of the simulations and the experimentally observed microstructures. This increases the reliability of this study. The herein acquired data are representative and have the perspective to allow researchers to understand the features of structure formation and predict the microstructures and properties of alloys of the considered system based on the results of phase composition modeling. Moreover, the results further aim to deepen the understanding of the possibility of using aluminum scrap as the raw material for the production of aluminum alloys. They can be used in planning alloying of aluminum alloys that are heavily enriched with iron and silicon, as well as for identifying the microstructure of real alloys. Last but not least, the prospects of this study are also in the applicability of the results to a wide range of alloys and flexible modeling conditions in the ThermoCalc software package.

## Figures and Tables

**Table 1:** Impurity elements content in commercial aluminum alloys (in wt.%).

System	Si	Fe	Cu	Mn	Mg	Cr	Zn	Ni	Ti	V	Zr	Be	Al
Aluminum	0.3	0.4	0.2	0.1	0.05	0.01	0.1	-	0.15	0.05	-	0.05	bal.
Al-Cu-Mg	3	1.5	7	1	2.7	0.1	1	1.4	0.2	0.15	0.25	-	bal.
Al-Cu-Mn	3	1.5	7	1	2.7	0.1	1	1.4	0.2	0.15	0.25	-	bal.
Al-Mn	0.6	0.8	0.3	1.5	1.3	0.2	0.4	-	0.05	0.05	0.05	0.0003	bal.
Al-Si	13	0.8	3	0.6	2	0.2	0.5	1.3	0.2	-	-	-	bal.
Al-Mg	0.5	0.7	0.2	1.2	7	0.35	1	0.05	0.2	0.02	0.2	0.005	bal.
Al-Mg-Si	1.3	0.8	1.4	1	1.4	0.35	0.8	0.2	0.2	-	0.12	0.005	bal.
Al-Zn-Mg	1.5	1	5.9	1	3	0.25	9	0.2	0.1	-	0.22	0.005	bal.
Al-Fe	1.5	2	0.3	1	0.1	0.05	0.1	-	0.1	-	0.45	-	bal.
Al-Li	0.3	0.3	5.8	0.8	0.05	Li 1.4	0.1	-	0.15	-	-	-	bal.

In accordance with additional instructions, individual brands may, at the customer’s request, contain elements such as lanthanum 0.1, cerium 0.01, cadmium 0.00005, gallium 0.03, boron 0.05, scandium 0.27, bismuth 0.6, lead 0.6, scandium 0.5, calcium 0.1, and molybdenum 0.06 (all in wt.%).

**Table 2:** Alloys compositions (in wt.%).

Alloy	Si	Fe	Cu	Mg	Mn	Cr	Ni	Zn	Ti	V	Zr	Be	Al
Alloy 0	7	33											Bal.
Alloy 1	7	33	4.2	-	-	-	-	-	-	-	-	-	Bal.
Alloy 2	7	33	-	0.84	-	-	-	-	-	-	-	-	Bal.
Alloy 3	7	33	-	-	0.9	-	-	-	-	-	-	-	Bal.
Alloy 4	7	33	-	-	-	0.21	-	-	-	-	-	-	Bal.
Alloy 5							0.84	-					
Alloy 6	7	33	-	-	-	-	-	5.4	-	-	-	-	Bal.
Alloy 7	7	33	-	-	-	-	-	-	0.12	-	-	-	Bal.
Alloy 8	7	33	-	-	-	-	-	-	-	0.09	-	-	Bal.
Alloy 9	7	33	-	-	-	-	-	-	-	-	0.27	-	Bal.
Alloy 10	7	33	-	-	-	-	-	-	-	-	-	0.003	Bal.
Alloy 11	7	33	4.2	0.84	0.9	0.21	0.84	5.4	0.12	0.09	0.27	0.003	Bal.
Alloy 12	5.18	33.6			1.57		0.19						Bal.
Alloy 13	3.72	29.5	0.02		0.18		0.02						Bal.

**Table 3:** Total impact of impurity/alloying elements on thermodynamic characteristics of the alloy.

Alloy	Liquidus Temp.	Solidus Temp.	Upper Boundary of the α_h_ Phase	Lower Boundary of the α_h_ Phase	The α_h_ Phase Content (Temp.)	Alloying Element, Amount
Alloy 0	1077	629	769	446	88.6(625) 93.1(465)	Fe_33_Si_7_
Alloy 1	1075	665	665	447	26(650) 21(465)	Cu, 4.2
Alloy 2	1060	600	720	446	86(594) 85(446)	Mg, 0.84
Alloy 3	1070	680	762	450	63(643) 63(450)	Mn, 0.9
Alloy 4	1070	629	769	446	80(625) 74(447)	Cr, 0.21
Alloy 5	1070	641	753	446	81(642) 75(446)	Ni, 0.84
Alloy 6	1110	399	650	462	41(462)	Zn, 5.4
Alloy 7	1070	630	767	446	88(624) 92(450)	Ti, 0.12
Alloy 8	1070	629	767	446	92(590) 92.7(458)	V, 0.09
Alloy 9	1070	629	769	446	89(620) 90.6(450)	Zr, 0.27
Alloy 10	1070	620	768	446	90.7(620) 92.6 (450)	Be, 0.003
Alloy 11	1110	533	-	-	-	All the elements considered
Alloy 12 experimental	1110	690	757	450	23(750) 58 (500)	Fe_33.59_, Si_5.18_, Mn_1.574_, Ni_0.19_
Alloy 13 experimental	1080	628	740	420	10(700) 73.3(470)	Fe_29.5_, Si_3.72_, Mn_0.18_, Ni_0.02,_ Cu_0.02_

**Table 4:** Summary of mechanical properties for both experimentally prepared alloys.

Alloy	Microhardness (HV)	Compressive Strength (MPa)	Elongation at Max. Strength (%)
Alloy 12	128 ± 6 (FCC Al) 756 ± 12 (intermetallics)	52 ± 2	10
Alloy 13	133 ± 6 (FCC Al) 450 ± 11 (intermetallics)	49 ± 2	11

## Data Availability

All data used are available in this article. Colored versions of the images are available in the online version of the article.
